# HLF regulates ferroptosis, development and chemoresistance of triple-negative breast cancer by activating tumor cell-macrophage crosstalk

**DOI:** 10.1186/s13045-021-01223-x

**Published:** 2022-01-06

**Authors:** Hengyu Li, Pinghua Yang, JingHan Wang, Jin Zhang, Qianyun Ma, Yingjie Jiang, Yani Wu, Tao Han, Daimin Xiang

**Affiliations:** 1 Department of Breast and Thyroid Surgery, Changhai Hospital, Naval Military Medical University, 230 Changhai Road, Shanghai, 200433 China; 2 Department of Hepatic Surgery, Third Affiliated Hospital of Naval Military Medical University, Shanghai, 200438 China; 3 Department of Hepatobiliary Surgery, East Hospital, School of Medicine, Tongji University, Shanghai, 200120 China; 4 Department of Urology Surgery, Changhai Hospital, Naval Military Medical University, Shanghai, 200433 China; 5 Department of Pathology, Changhai Hospital, Naval Military Medical University, Shanghai, 200433 China; 6 Department of Oncology, The First Affiliated Hospital of China Medical University, 155 Nanjing North Street, Shenyang, 110001 China; 7 State Key Laboratory of Oncogenes and Related Genes, Shanghai Cancer Institute, Renji Hospital, Shanghai Jiao Tong University School of Medicine, No. 25, Lane 2200, Xietu Road, Shanghai, 200127 China

**Keywords:** Triple-negative breast cancer, Tumor-associated macrophages, TGF-β1/SMAD3/HLF/IL-6/JAK2/STAT3 pathway, GGT1, Ferroptosis

## Abstract

Tumor-associated macrophages (TAMs) are major components of the tumor microenvironment (TME) which are closely associated with the tumor malignant progression. However, the regulatory mechanisms by which TAMs influence the progression of triple-negative breast cancer (TNBC) remain unclear. Here, we report that hepatic leukemia factor (HLF) acts as a novel oncoprotein in TNBC. We found that HLF was regulated by transforming growth factor-beta1 (TGF-β1) that is secreted by TAMs. Then, HLF transactivated gamma-glutamyltransferase 1 (GGT1) to promote the ferroptosis resistance, thus driving TNBC cell proliferation, metastasis and cisplatin resistance. Reciprocally, IL-6 produced by TNBC cells activated the JAK2/STAT3 axis to induce TGF-β1 secretion by TAMs, thus constituted a feed-forward circuit. The accuracy of TNBC patient prognosis could be improved by employing a combination of HLF and GGT1 values. Thus, our findings document that the interactive dialogue between TNBC cells and TAMs promotes sustained activation of HLF in tumor cells through the IL-6-TGF-β1 axis. Subsequently, HLF promotes the ferroptosis resistance in TNBC cells via GGT1 and ultimately facilitates the malignant tumor progression. Our study provides a potential target for the treatment of TNBC.

**Supplementary Information:**

The online version contains supplementary material available at 10.1186/s13045-021-01223-x.

**To the editor,**

TAMs are major components of the tumor microenvironment that directly affect tumor cell growth, neoangiogenesis and immunosuppression [1–3]. The crosstalk between tumor cells and TAMs has been studied in mammary tumors [4]; however, the mechanisms underlying the activation of TAMs by tumor cells remain unclear in TNBC.

Here, we found that HLF, which has been reported as an important regulator in liver fibrosis and hepatocellular carcinoma [5, 6], was not only increased in TNBC samples but also associated with poor outcome in TNBC patient cohort (Fig. 1a, b, Additional file 1: Fig. S1, Additional file 3: Table S1). Bioinformatics analysis of the HLF promoter revealed a set of potential binding sites for different transcription factors including SMAD3. HLF expression in TNBC cells was decreased by interference with SMAD3 (Fig. 1c, d, Additional file 1: Fig. S2a–e). Moreover, we found that TGF-β1 secreted by TAMs induced HLF expression in TNBC (Fig. 1e, Additional file 1: Fig. S2f–j). ChIP analysis revealed that SMAD3 binds to the HLF promoter (Fig. 1f). Nevertheless, the HLF-Mut luciferase reporter could not be activated by SMAD3 (Additional file 1: Fig. S2k).

**Fig. 1:**
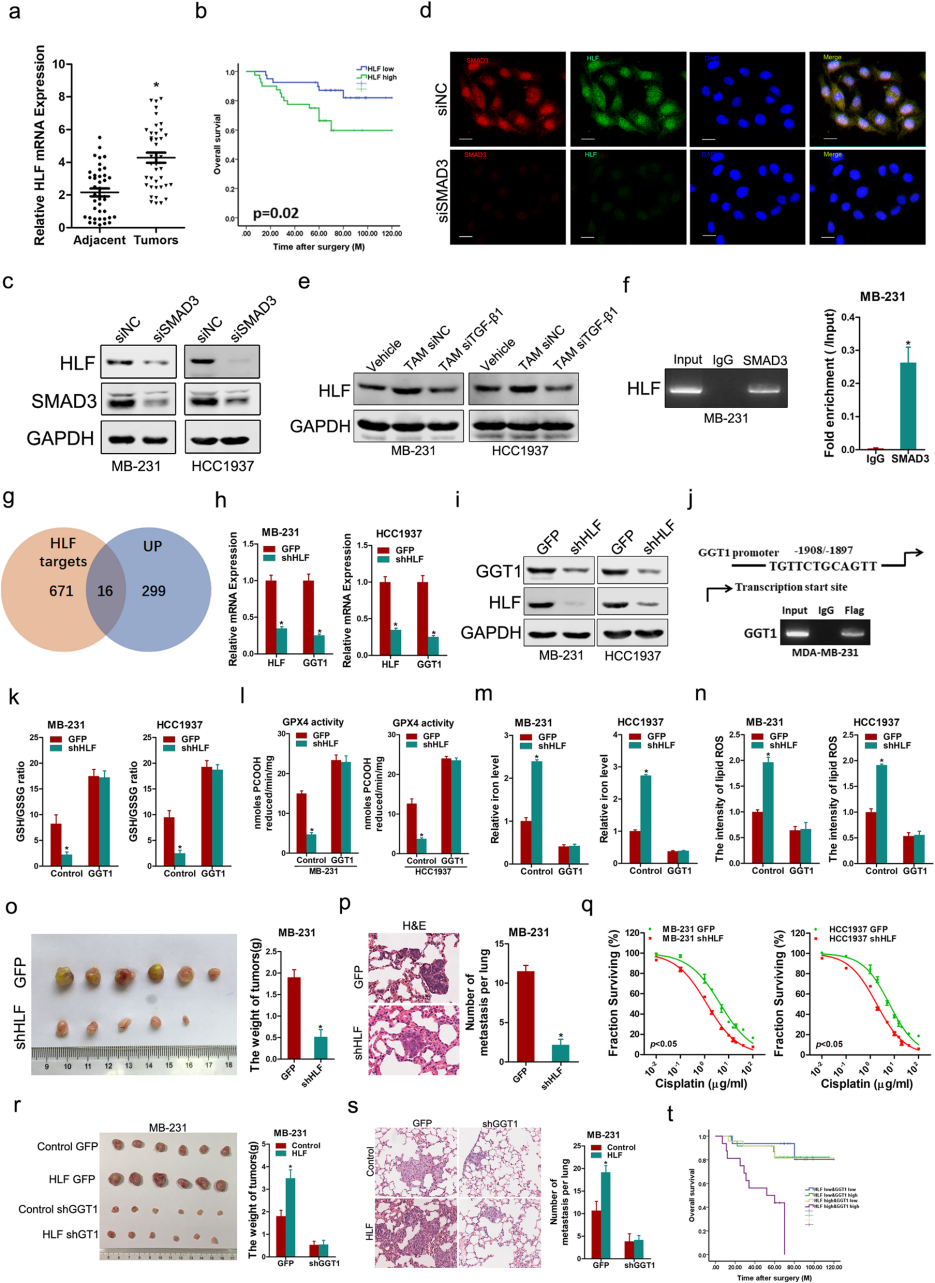
TGF-β1/SMAD3-transactived HLF promotes TNBC ferroptosis resistance, proliferation, metastasis and chemoresistance through GGT1 signaling. **a** Real-time PCR analysis of HLF in 40 pairs of TNBC samples and their corresponding peritumoral normal samples. **b** The overall survival time after surgery of the patients in TNBC patients was compared between the “HLF low, *n* = 40” and “HLF high, *n* = 40” groups. **c** MB-231/HCC1937 cells were transfected with siSMAD3 followed by western blot analysis. **d** MB-231 transfected with siSMAD3 or siNC was subjected to dual immunofluorescence staining. Representative images are shown. The nuclei were counterstained with 4′,6-diamidino-2-phenylindole (DAPI). Scale bar = 20 μm. **e** TAMs were transfected with siTGF-β or siNC. MB-231/HCC1937 cells were cocultured with TAMs siTGF-β or TAMs siNC followed by western blot analysis. **f** MB-231 cells subjected to ChIP assay with anti-SMAD3 or anti-IgG antibody. **g** Venn diagram representing the overlap between HLF targets, identified by ChIP-seq, and RNA-seq. **h** Real-time PCR analysis of the mRNA expression of HLF and GGT1 in shHLF or control TNBC cells. **i** Western blot analysis of the protein expression of HLF and GGT1 in shHLF or control TNBC cells. **j** MB-231 cells subjected to ChIP assay with anti-Flag or anti-IgG antibody. **k** The ratio of reductive GSH to oxidative GSH was measured by the GSH/GSSG quantification kit in the indicated cells. **l** Substantially lower GPX4-specific activity was detected in the indicated cells using PCOOH as a substrate. **m** Total iron in the indicated cells was analyzed using Iron Assay Kit. **n** Oxidative stress in the indicated cells was assessed by the levels of lipid ROS. **o** MB-231 shHLF or control cells were subcutaneously injected into nude mice (n = 6) for xenograft assay. Tumor average weight in each group was shown. **p** Lung H&E staining of nude mice inoculated MB-231 shHLF and control cells via tail vein for 12 weeks. The number of lung metastatic foci in each group (*n* = 7) were also calculated, *p < 0.05. **q** Cell survival curves of shHLF and control TNBC cells treated with cisplatin with a dose escalation from 0 to 100 μg/ml. Data are presented as mean ± SD (*n* = 4) from one of three independent experiments. **r** Representative xenograft images at 6 weeks after the injection of indicated TNBC cells into nude mice. The weight of xenografts in each group (*n* = 6) was calculated. **s** Representative microscopic images of pulmonary metastatic lesions at 12 weeks after the injection of indicated TNBC cells into the tail vein of nude mice. The number of lung metastatic tumors in each group (*n* = 6) was calculated. **t** Kaplan–Meier analysis of overall survival of TNBC patients with high or low HLF and GGT1. *p* < 0.001. All results are presented as the mean ± SD, and statistical significance was assessed using a two-tailed Student’s t test. *p < 0.05

Mechanistically, GGT1 transcription was downregulated in HLF-knockdown cells while increased in HLF overexpression cells, respectively (Fig. 1g–i, Additional file 1: Fig. S3a–e). There was a close correlation between HLF levels and GGT1 expression in TNBC tumor specimens (Additional file 1: Fig. S3f). Significant enrichment of HLF in the promoter region of *GGT1* was detected by ChIP assay (Fig. 1j). Nevertheless, the GGT1-Mut luciferase reporter could not be suppressed by HLF interference (Additional file 1: Fig. S3g).

GGT1 catalyzes the cleavage of extracellular GSH into its components to provide cysteine for the production of intracellular GSH [7]. As expected, the GSH/GSSG ratio was decreased in HLF-knockdown cells (Additional file 1: Fig. S4a). A reduction in the GSH/GSSG ratio causes deactivation of ferroptosis regulator GPX4 [8]. Accordingly, the GPX4 activity was downregulated in HLF-knockdown cells (Additional file 1: Fig. S4b). Meanwhile, the iron, MDA and lipid ROS levels were increased in HLF-knockdown cells (Additional file 1: Fig. S4c–e). Notably, the GSH/GSSG ratio and deactivation of GPX4 in HLF-knockdown TNBC cells could be restored through introduction of GGT1 (Fig. 1k, l). Moreover, the HLF depletion-enhanced ferroptosis was reduced by GGT1 overexpression in TNBC cells (Fig. 1m, n, Additional file 1: Fig. S4f). Taken together, these data indicate that HLF inhibits ferroptosis by regulating the GGT1/GSH/GPX4 axis in TNBC cells.

GGT1, an enzyme localized and bound to the plasma membrane, has been reported to be involved in the regulation of tumorigenesis [9]. Functionally, the cellular proliferation and metastasis were significantly decreased in HLF-knockdown cells in vitro and in vivo (Fig. 1o, p, Additional file 1: Fig. S5a–g). Also, the sensitivity of TNBC cells to cisplatin was increased on stable knockdown of HLF (Fig. 1q, Additional file 1: Fig. S5h–j). Importantly, our data showed that the interference of GGT1 blocked the cellular proliferation, metastasis and cisplatin resistance induced by HLF (Fig. 1r, s, Additional file 1: Fig. S6a–e). Furthermore, HLF depletion-mediated proliferation ability, invasive capacity and cisplatin sensitivity could be recovered by treatment with the ferroptosis inhibitor liproxstatin-1 (Additional file 1: Fig. S6f–j). Clinical investigation revealed that TNBC patients with both elevated HLF and GGT1 predict a worse prognosis (Fig. 1t).

Human Cytokine Antibody Array (Additional file 2) showed that six cytokines were significantly increased in the conditioned medium (CM) of MB-231 HLF cells (fold-change > 2) (Additional file 1: Fig. S7a). Gene expression analysis further showed that of IL-6 was the most significantly altered (Fig. 2a, b, Additional file 1: Fig. S7b–e). The activation of IL-6 promoter can be attenuated through HLF knockdown, or enhanced via HLF overexpression, respectively (Additional file 1: Fig. S7f, g). Furthermore, a putative homologous HLF binding site within the human IL-6 promoter was detected by ChIP assays (Fig. 2c). As expected, THP-1 cells treated with IL-6 or CM from TNBC control cells became stretched and elongated (Fig. 2d).

**Fig. 2:**
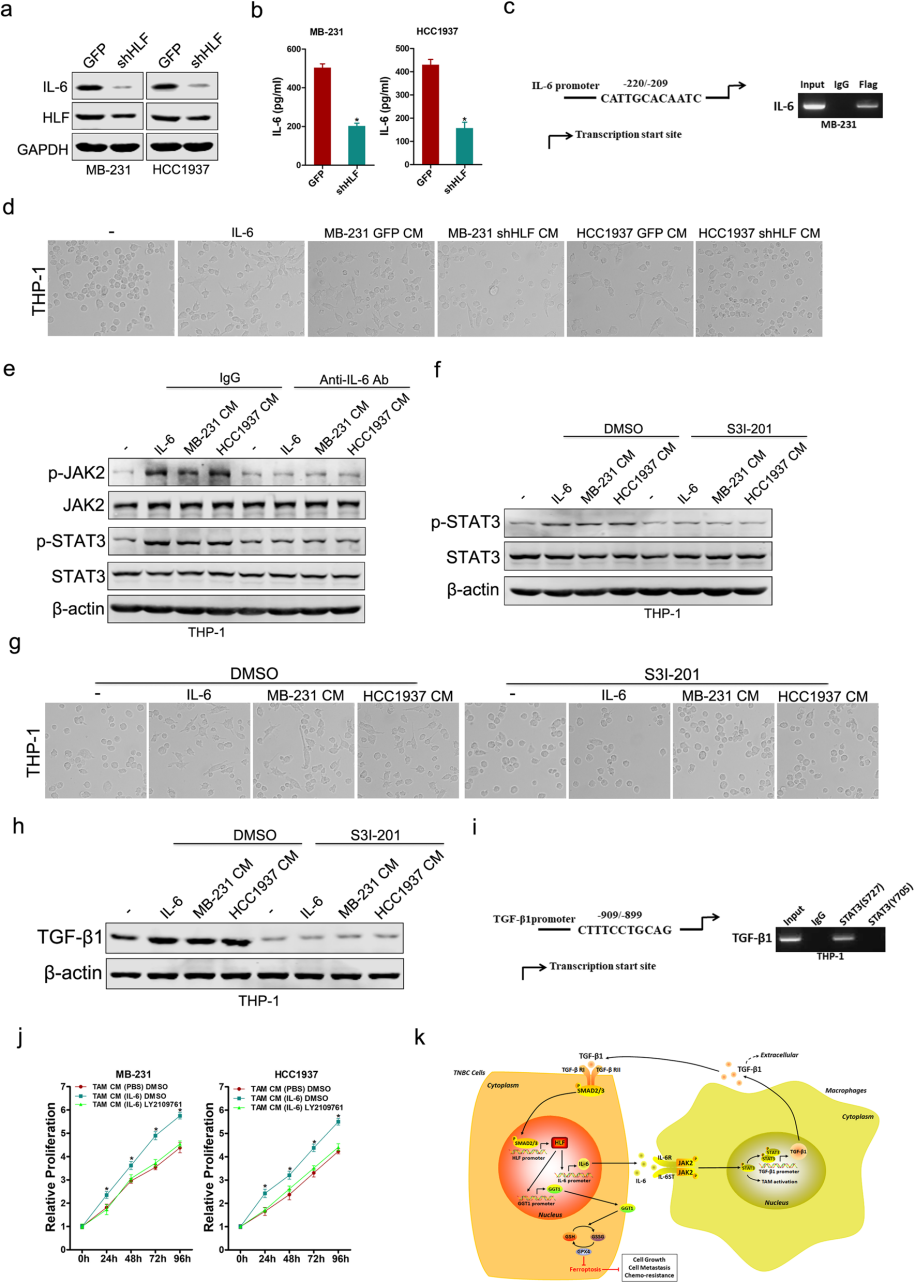
TNBC-derived IL-6 activates macrophage to a TAM-like phenotype via JAK2/STAT3 axis. **a** Western blot analysis of the protein expression of HLF and IL-6 in shHLF or control TNBC cells. **b** The IL-6 secreted from the shHLF or control TNBC cells were quantified by ELISA using the anti-IL-6 mAb. **c** MB-231 cells subjected to ChIP assay with anti-Flag or anti-IgG antibody. **d** THP-1 cells were cultured with IL-6 or indicated CM for 48 h. The representative bright-field images of macrophages treated by the respective conditioned media are shown (magnification, × 200). **e** Expression of phospho-JAK2 and phospho-STAT3 in THP-1 cells treated with IL-6, TNBC CM in the presence or absence of control IgG or an IL-6 neutralizing antibody. **f** Expression of phospho-STAT3 in THP-1 cells without or with the coculture of IL-6, TNBC CM alone or with S3I-201. **g** Morphology of THP-1 cells without or with the coculture of IL-6, TNBC CM alone or with S3I-201. **h** Western blot analysis of TGF-β1 in THP-1 cells without or with the coculture of IL-6, TNBC CM alone or with S3I-201. **i** THP-1 cells subjected to ChIP assay with anti-phospho-STAT3 or anti-IgG antibody. **j** CCK-8 analysis of TNBC cells treated with CM of control TAMs, IL-6-activated TAMs alone or with LY2109761. **k** The schematic model of the mechanism underlying the role of HLF in TNBC ferroptosis resistance, proliferation, metastasis and chemoresistance. All results are presented as the mean ± SD, and statistical significance was assessed using a two-tailed Student’s t test. *p < 0.05

Mechanistically, we found that stimulation of THP-1 cells with IL-6 or CM from TNBC cells resulted in increased expression of p-JAK2 and p-STAT3, whereas treatment with IL-6 neutralizing antibodies or S3I-201 inhibited this pathway (Fig. 2e–g, Additional file 1: Fig. S8a–d). Moreover, the expression of TGF-β1 was significantly increased in THP-1 cells cocultured with IL-6 or CM from TNBC cells, whereas treatment with IL-6 neutralizing antibodies or S3I-201 inhibited it (Fig. 2h, Additional file 1: Fig. S8e–g). A putative homologous STAT3(S727) but not STAT3(Y705) binding site within the human TGF-β1 promoter was identified by ChIP assays (Fig. 2i). Nevertheless, the TGF-β1-Mut luciferase reporter could not be suppressed by STAT3 interference (Additional file 1: Fig. S8h).

The supernatants from TAMs pre-treated with IL-6, which contained multiple pro-inflammatory cytokines and chemokines as previous study [10], enhanced the HLF-GGT1 axis, increased the GSH/GSSG ratio and amplified GPX4 activity of TNBC cells, which associated with the suppression of ferroptosis (Additional file 1: Fig. S9a–g). In addition, CM of TAMs pre-treated with IL-6 not only promoted proliferation and metastasis of TNBC cells but also reduced their sensitivity to cisplatin (Fig. 2j, Additional file 1: Fig. S9h–l), and these phenomena were partially reversed by TGF-β receptor inhibitor LY2109761. These results indicate that TAMs educated by IL-6 can promote the ferroptosis resistance, progression and chemoresistance of TNBC cells.

In summary, we identified that TAM-derived TGF-β1 induced ferroptosis resistance in TNBC cells and hence enhanced progression and chemoresistance by regulating the SMAD3/HLF/GGT1/GPX4 pathway. Reciprocally, elevated IL-6 expression by TAM-educated TNBC cells significantly promoted the recruitment of macrophages via JAK2/STAT3 axis in a feedback way (Fig. 2k).

## Supplementary Information

**Additional file 1:** Supplementary Figures.

**Additional file 2:** Supplementary Methods.

**Additional file 3:** Supplementary Tables.

## Data Availability

All supporting data are included in the manuscript and supplemental files. Additional data are available upon reasonable request to the corresponding author.

## Abbreviations

TAMs: Tumor-associated macrophages
TME: Tumor microenvironment
HLF: Hepatic leukemia factor
TNBC: Triple-negative breast cancer
TGF-β1: Transforming growth factor-beta1
GGT1: Gamma-glutamyltransferase 1
HCC: Hepatocellular carcinoma
TMA: Tissue microarrays
ChIP: Chromatin immunoprecipitation
CCK-8: Cell Counting Kit-8
EdU: 5-Ethynyl-20-deoxyuridine
GSH: Glutathione
DAPI: 4′,6-Diamidino-2-phenylindole
CM: Conditioned medium
