# Supplementary material for: HLF regulates ferroptosis, development and chemoresistance of triple-negative breast cancer by activating tumor cell-macrophage crosstalk
Source: J Hematol Oncol. 2022 Jan 6;15:2. doi: 10.1186/s13045-021-01223-x (PMC8740349; doi:10.1186/s13045-021-01223-x)
Supplement: Supplementary file 1 — Additional file 1: Supplementary Figures. [file 13045_2021_1223_MOESM1_ESM.doc]

**Additional file 1**

**
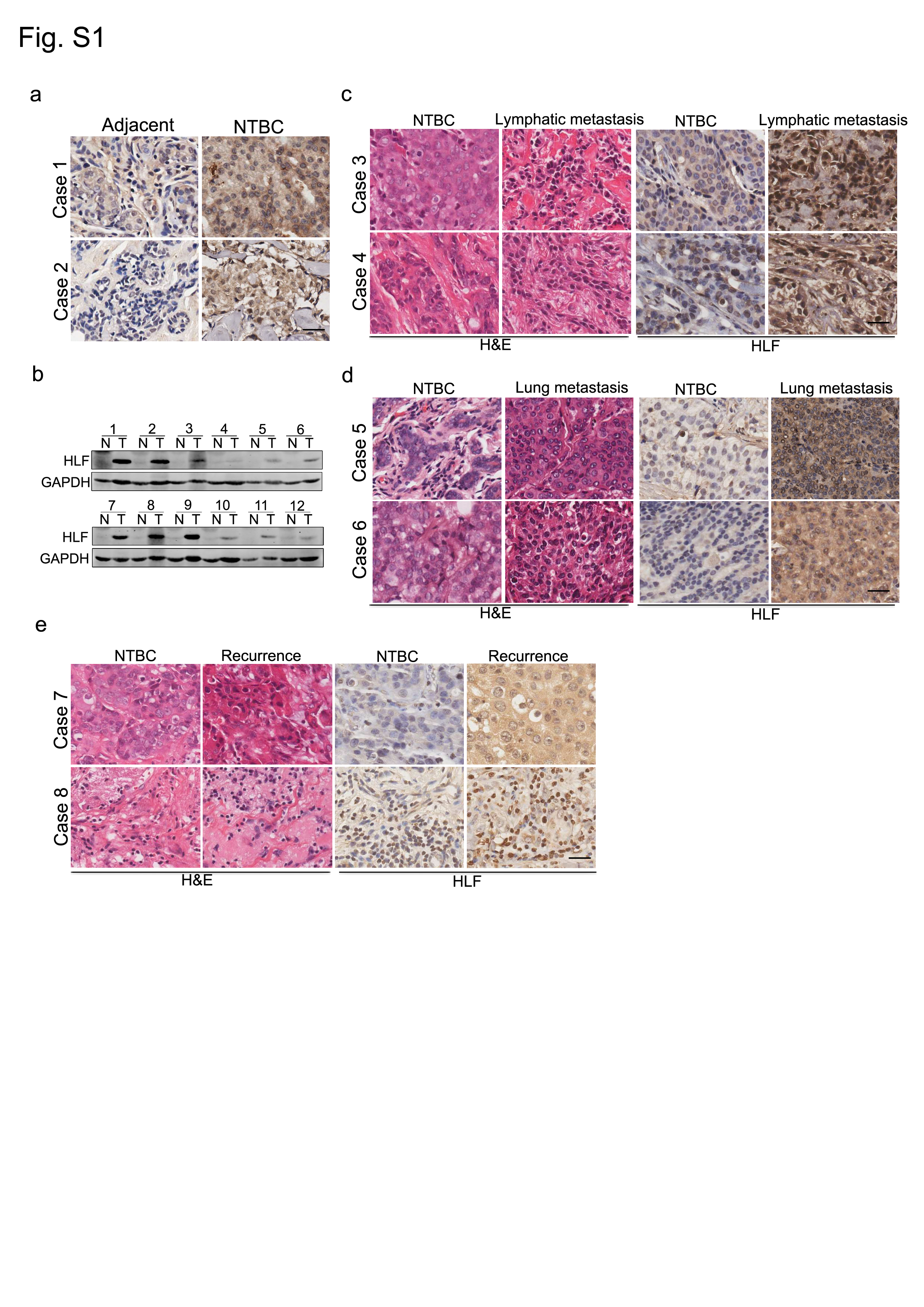
**

**Fig. S1. Expression of HLF is elevated in human TNBC tissues.**

**a** Representative images of IHC staining of HLF in matched human peritumoral normal and TNBC tissues. Scale bar=25 μm.

**b** Western blot analysis of HLF levels in 12 pairs of TNBC samples and their corresponding cancerous samples.

**c** Representative images of H&E and IHC staining of HLF in matched human TNBC and lymph node metastasis tissues. Scale bar=25 μm.

**d** Representative images of H&E and IHC staining of HLF in matched human TNBC and pulmonary metastasis tissues. Scale bar=25 μm.

**e** Representative images of H&E and IHC staining of HLF in matched human TNBC and relapsing TNBC tissues. Scale bar=25 μm.

**
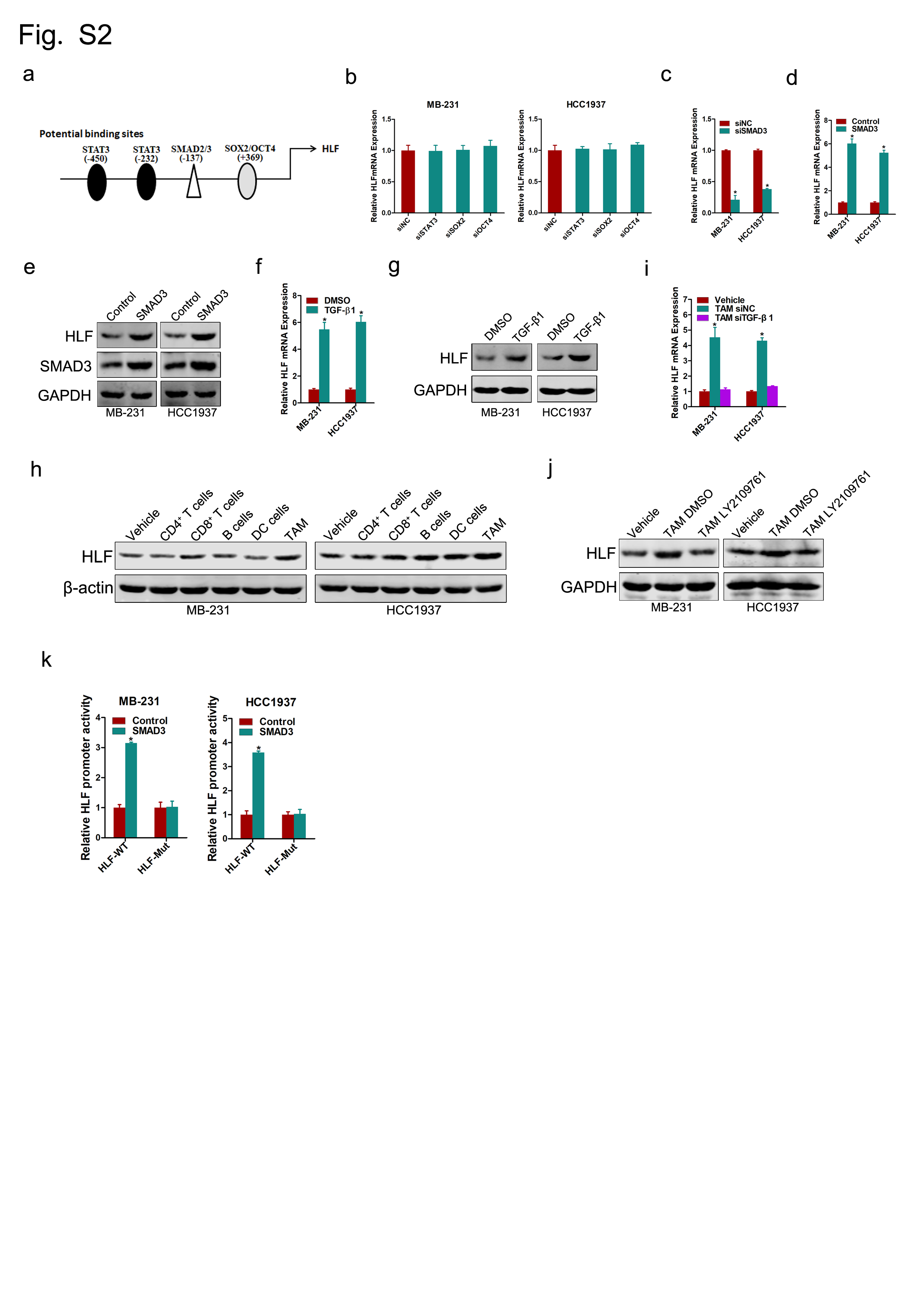
**

**Fig. S2. HLF is activated by TGF-β/SMAD2/3 in TNBC.**

**a** The potential binding sites within the promoter region of HLF.

**b** MB-231/HCC1937 cells were transfected with siSTAT3, siSOX2, siOCT4, or siNC for 24 hours followed by real-time PCR analysis.

**c** MB-231/HCC1937 cells were transfected with siSMAD3 followed by real-time PCR analysis.

**d** MB-231/HCC1937 cells were infected with SMAD3 overexpression virus followed by real-time PCR analysis.

**e** MB-231/HCC1937 cells were infected with SMAD3 overexpression virus followed by western blot analysis.

**f** MB-231/HCC1937 cells were treated with TGF-β1 followed by real-time PCR analysis.

**g** MB-231/HCC1937 cells were treated with TGF-β1 followed by western blot analysis.

**h** MB-231/HCC1937 cells were cocultured with CD4^+^ T cells, CD8^+^ T cells, B cells, dendritic cells (DC cells) or TAMs followed by western blot analysis.

**i** TAMs were transfected with siTGF-β1 or siNC. MB-231/HCC1937 cells were cocultured with TAMs siTGF-β1 or TAMs siNC followed by real-time PCR analysis.

**j** MB-231/HCC1937 cells, cocultured with TAMs, were treated with LY2109761 or not followed by western blot analysis.

**k** The luciferase activity of the HLF-WT or HLF-Mut promoter was measured in SMAD3 knockdown and control TNBC cells, and the relative activity was presented (relative to control).

All results are presented as the mean ± SD, and statistical significance was assessed using a two-tailed Student t test. *p < 0.05.

**
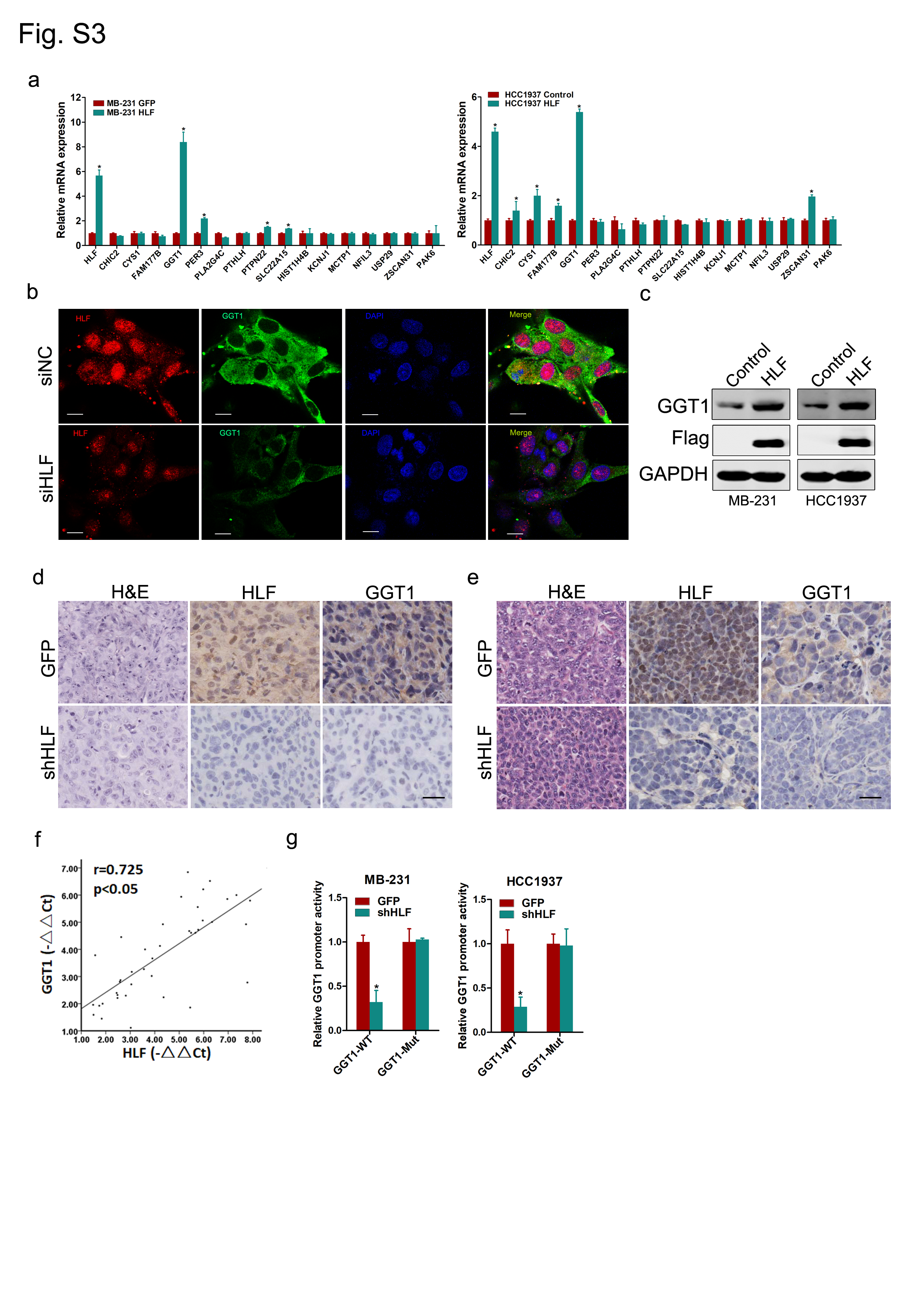
**

**Fig. S3. HLF transcriptionally activates GGT1 in TNBC cells.**

**a** HLF overexpression and control TNBC cells were subjected to real-time PCR analysis.

**b** Representative images of dual immunofluorescence staining of HLF and GGT1 in siHLF or control TNBC cells. The nuclei were counterstained with 4’, 6-diamidino-2-phenylindole. Scale bar=20 μm.

**c** HLF overexpression and control TNBC cells were subjected to western blot analysis.

**d** Representative images of H&E and IHC staining of HLF and GGT1 in xenografted tumors formed by MB-231 shHLF or control cells. Scale bar=25 μm.

**e** Representative images of H&E and IHC staining of HLF and GGT1 in liver metastasis lesions formed by MB-231 shHLF or control cells. Scale bar=25 μm.

**f** The correlation between HLF levels and GGT1 expression in TNBC was evaluated in a group of 40 TNBC specimens using Pearson’s correlation analysis.

**g** The luciferase activity of the GGT1-WT or GGT1-Mut promoter was measured in shHLF and control TNBC cells, and the relative activity was presented (relative to control).

All results are presented as the mean ± SD, and statistical significance was assessed using a two-tailed Student t test. *p < 0.05.


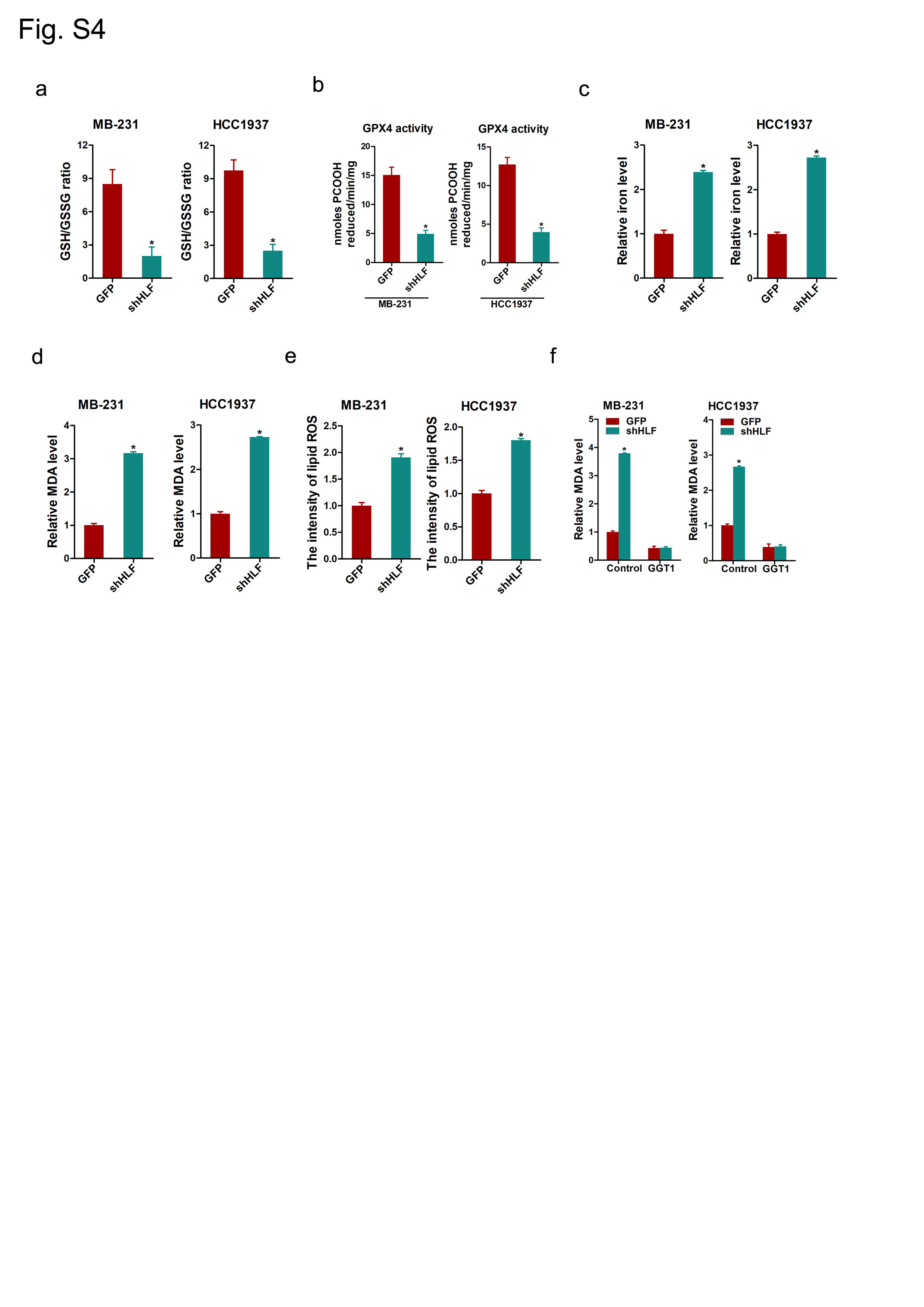


**Fig. S4. HLF inhibits ferroptosis via targeting the GGT1/GSH/GPX4 axis in TNBC cells.**

**a** The ratio of reductive GSH to oxidative GSH was measured by the GSH/GSSG quantification kit in shHLF and control TNBC cells.

**b** Substantially lower GPX4-specific activity was detected in shHLF and control TNBC cells using PCOOH as a substrate.

**c** Total iron in shHLF and control TNBC cells were analyzed using Iron Assay Kit.

**d, e** Oxidative stress in shHLF and control TNBC cells was assessed by the levels of MDA and lipid ROS.

**f** Oxidative stress in the indicated cells was assessed by the levels of MDA.

All results are presented as the mean ± SD, and statistical significance was assessed using a two-tailed Student t test. *p < 0.05.


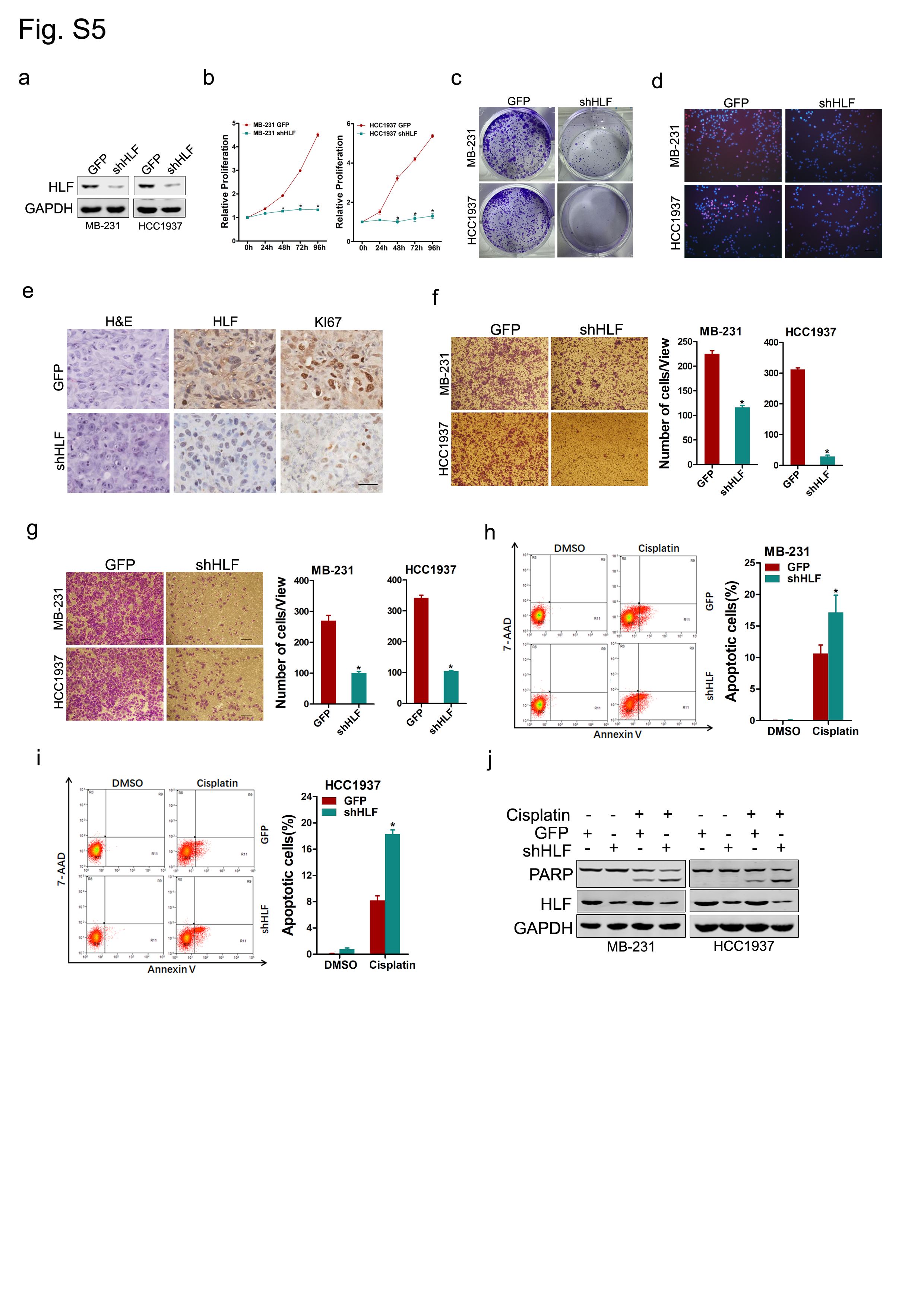


**Fig. S5. HLF drives TNBC growth, metastasis and chemoresistance.**

**a** MB-231/HCC1937 cells were transfected with shHLF or control virus, and then subjected to western blot analysis.

**b** Proliferation of shHLF and control TNBC cells was evaluated by determining the cell viability by Cell Counting Kit 8 Assay.

**c** shHLF or control TNBC cells were subjected to the colony growth assay. The colonies formed were fixed and stained with crystal violet, and representative images were shown.

**d** Representative images of EdU staining of proliferating shHLF or control TNBC cells. EdU^+^ cells were stained with red immunofluorescence. The nuclei were counterstained with DAPI. Scale bar=50 μm.

**e** Xenograft tumors were excised and subjected to H&E and IHC staining six weeks post inoculation. Scale bar=25 μm.

**f** The migration properties of shHLF or control TNBC cells were analyzed using transwell assay. The cell counts are expressed as the mean number of cells per field of view.

**g** The invasive properties of shHLF or control TNBC cells were analyzed using Matrigel-coated Boyden chamber. The cell counts are expressed as the mean number of cells per field of view.

**h** MB-231 shHLF and control cells treated with cisplatin (4 µg/ml) for 48 hours were subjected to flow cytometry analysis.

**i** HCC1937 shHLF and control cells treated with cisplatin (4 µg/ml) for 48 hours were subjected to flow cytometry analysis.

**j** MB-231/HCC1937 shHLF and control TNBC cells treated with cisplatin (4 µg/ml) for 48 hours were subjected to western blot analysis.

All results are presented as the mean ± SD, and statistical significance was assessed using a two-tailed Student t test. *p < 0.05.


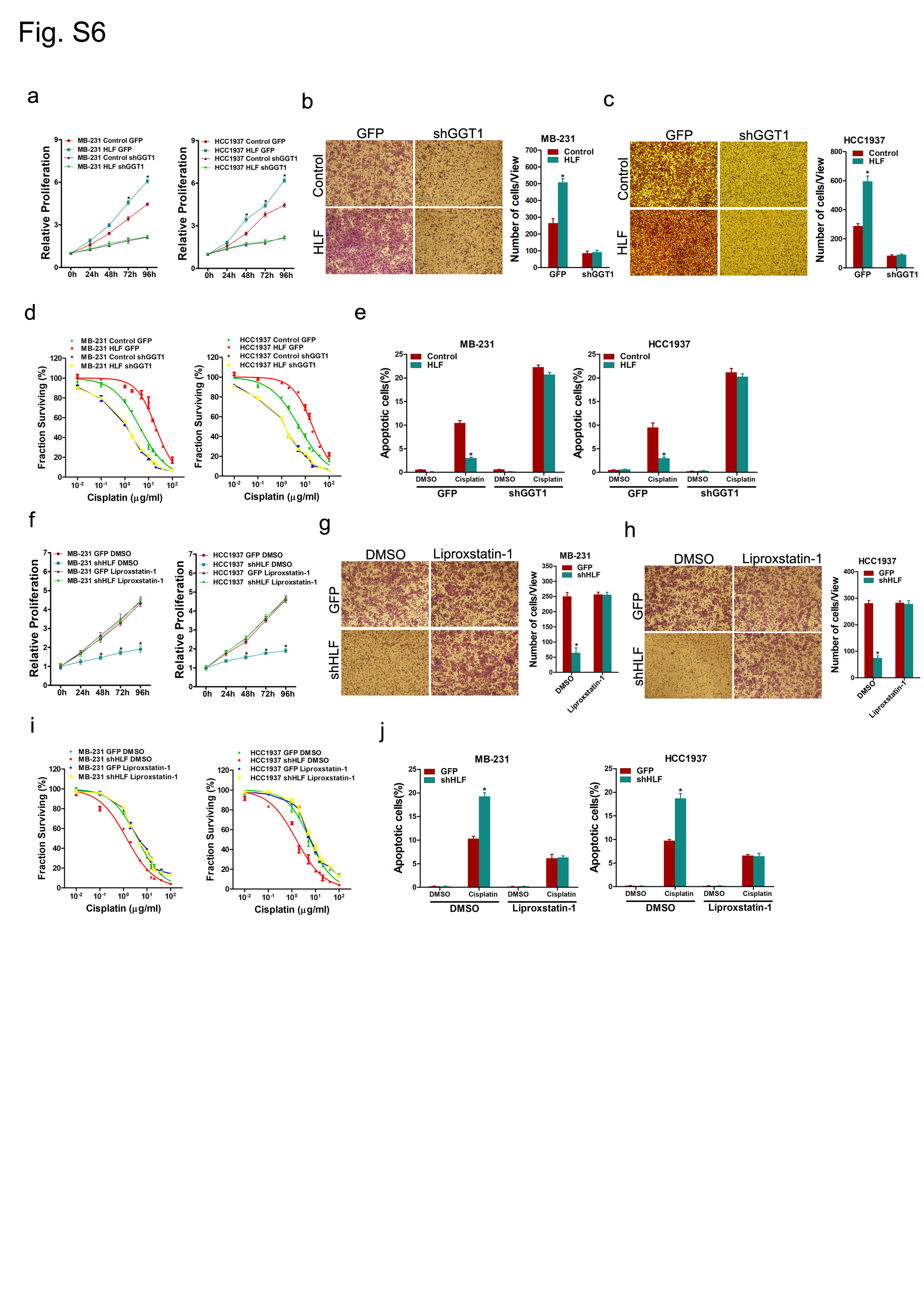


**Fig. S6. HLF activates GGT1 to promote TNBC progression and chemoresistance.**

**a** HLF overexpression and control TNBC cells were infected with shGGT1 virus or control virus, and then subjected to CCK8 assay.

**b, c** HLF overexpression and control cells were infected with shGGT1 virus or control virus, and then subjected to Invasion chamber assay.

**d** HLF overexpression and control TNBC cells were infected with shGGT1 virus or control virus. The cells were then treated with cisplatin and cell survival curves was examined.

**e** HLF overexpression and control TNBC cells were infected with shGGT1 virus or control virus. The cells were then treated with cisplatin (4 µg/ml) for 48 hours and their apoptosis was examined by flow cytometry.

**f** shHLF and control TNBC cells were treated with liproxstatin-1 or not, and then subjected to CCK8 assay.

**g, h** MB-231/HCC1937 shHLF and control TNBC cells were treated with liproxstatin-1 or not, and then subjected to Invasion chamber assay.

**i** shHLF and control TNBC cells were treated with liproxstatin-1 or not. The cells were then treated with cisplatin and cell survival curves was examined.

**j** shHLF and control TNBC cells were treated with liproxstatin-1 or not. The cells were then treated with cisplatin (4 µg/ml) for 48 hours and their apoptosis was examined by flow cytometry.

All results are presented as the mean ± SD, and statistical significance was assessed using a two-tailed Student t test. *p < 0.05.

**
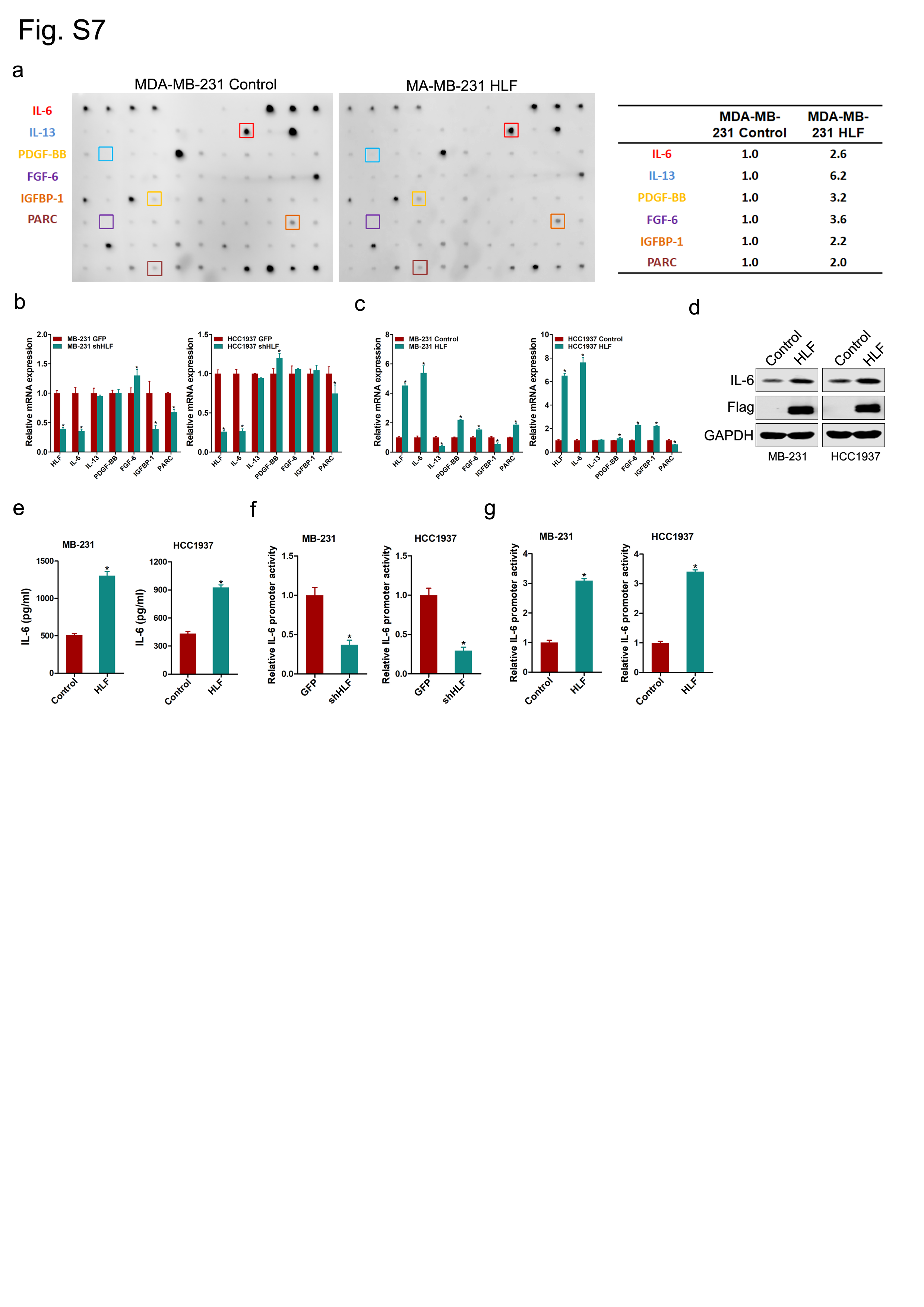
**

**Fig. S7. HLF transcriptionally activates IL-6 in TNBC cells.**

**a** Cytokine array of the CM of MB-231 Control cells and MB-231 HLF cells. A table summarizing the relative signal intensity of indicated cytokines is presented in the right corner.

**b** Real-time PCR analysis of the mRNA expression of a series of cytokines in shHLF or control TNBC cells.

**c** Real-time PCR analysis of the mRNA expression of a series of cytokines in HLF overexpression or control TNBC cells.

**d** Western blot analysis of the protein expression of HLF and IL-6 in HLF overexpression or control TNBC cells.

**e** The IL-6 secreted from the HLF overexpression or control TNBC cells were quantified by ELISA using the anti-IL-6 mAb.

**f** The luciferase reporter activity of IL-6 promoter was measured in shHLF or control TNBC cells.

**g** The luciferase reporter activity of IL-6 promoter was measured in HLF overexpression or control TNBC cells.

All results are presented as the mean ± SD, and statistical significance was assessed using a two-tailed Student t test. *p < 0.05.


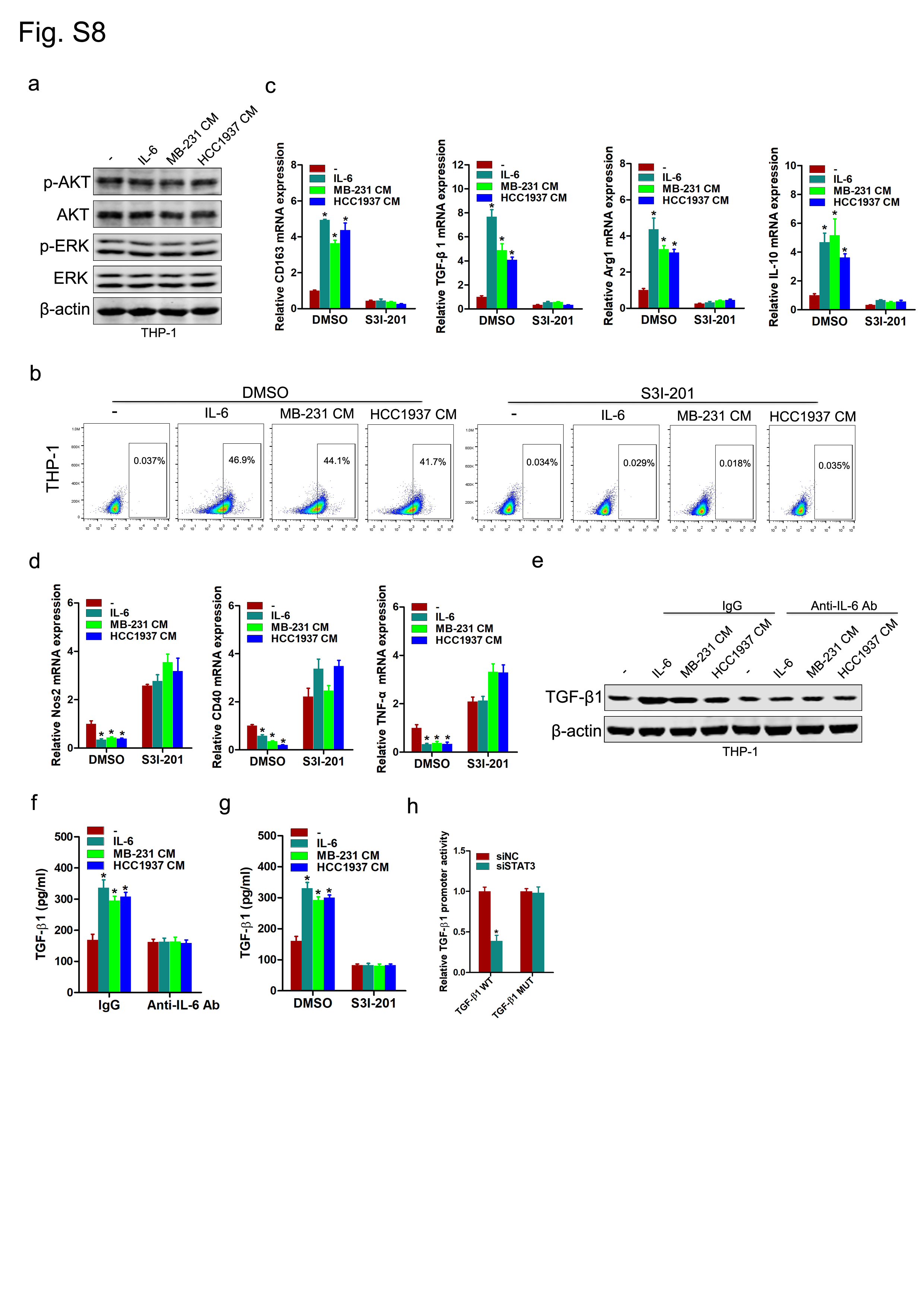


**Fig. S8. The JAK2/STAT3 axis contributes to IL-6-induced macrophage activation.**

**a** Expression of phospho-AKT and phospho-ERK in THP-1 cells treated with IL-6 or TNBC CM.

**b** Expression of CD163 in THP-1 cells without or with the coculture of IL-6, TNBC CM alone or with S3I-201.

**c** Real-time PCR analysis of CD163, TGF-β1, Arg1 and IL-10 in THP-1 cells without or with the coculture of IL-6, TNBC CM alone or with S3I-201.

**d** Real-time PCR analysis of Nos2, CD40 and TNF-α in THP-1 cells without or with the coculture of IL-6, TNBC CM alone or with S3I-201.

**e** Western blot analysis of TGF-β1 in THP-1 cells without or with the coculture of IL-6, TNBC CM alone or with IL-6 neutralizing antibody.

**f** ELISA analysis of TGF-β1 in THP-1 cells without or with the coculture of IL-6, TNBC CM alone or with IL-6 neutralizing antibody.

**g** ELISA analysis of TGF-β1 in THP-1 cells without or with the coculture of IL-6, TNBC CM alone or with S3I-201.

**h** The luciferase reporter activity of TGF-β1-WT or TGF-β1-Mut promoter was measured in STAT3 knockdown and control THP-1 cells, and the relative activity was presented (relative to control).

All results are presented as the mean ± SD, and statistical significance was assessed using a two-tailed Student t test. *p < 0.05.


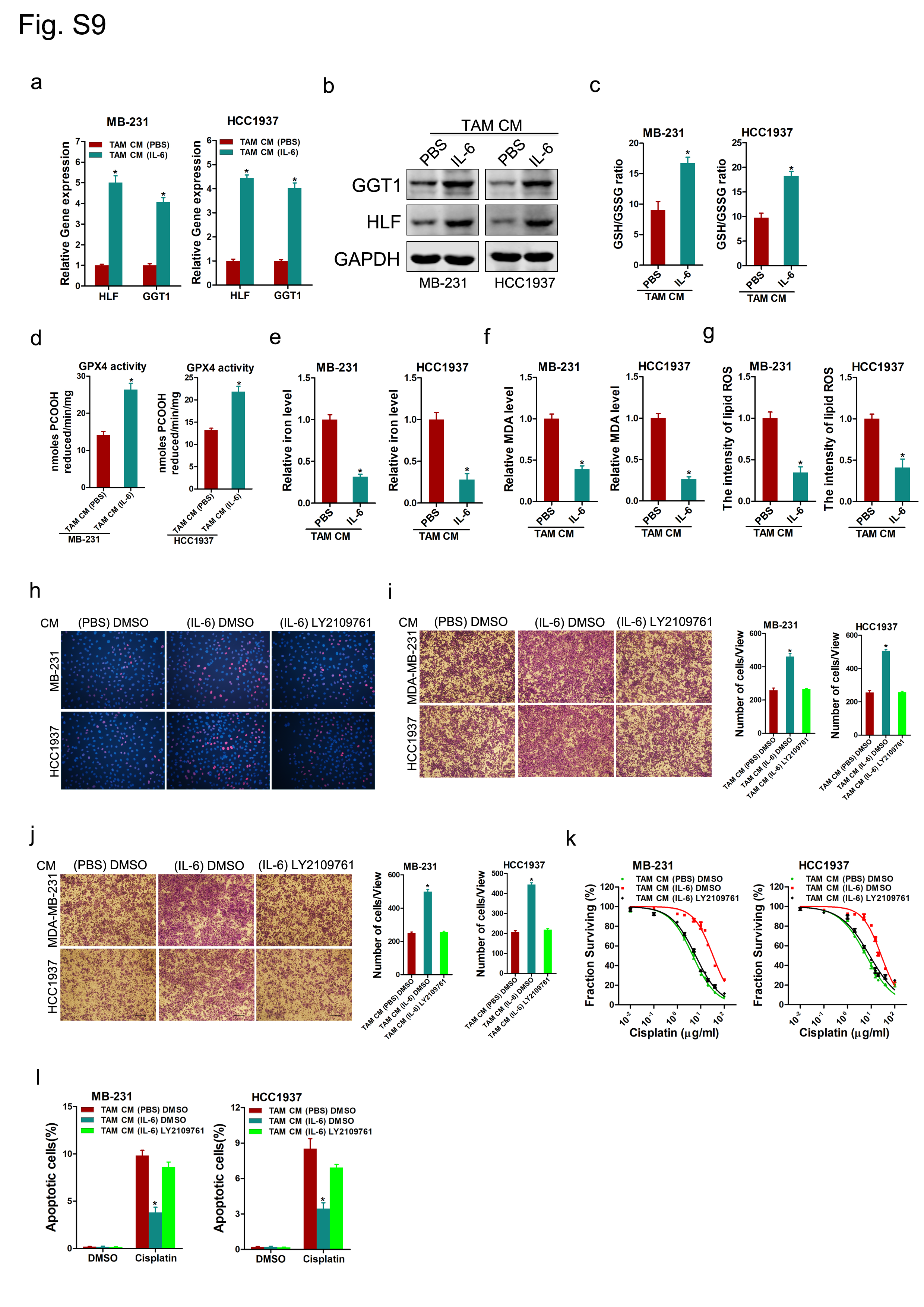


**Fig. S9. Activated TAMs promote TNBC ferroptosis resistance, proliferation, metastasis and chemoresistance.**

**a** Real-time PCR analysis of HLF and GGT1 in TNBC cells treated with CM of control TAMs or IL-6 activated TAMs.

**b** Western blot analysis of HLF and GGT1 in TNBC cells treated with CM of control TAMs or IL-6 activated TAMs.

**c** The ratio of reductive GSH to oxidative GSH was measured by the GSH/GSSG quantification kit in TNBC cells treated with CM of control TAMs or IL-6 activated TAMs.

**d** Substantially lower GPX4-specific activity was detected in TNBC cells treated with CM of control TAMs or IL-6 activated TAMs using PCOOH as a substrate.

**e** Total iron were analyzed using Iron Assay Kit in TNBC cells treated with CM of control TAMs or IL-6 activated TAMs.

**f, g** Oxidative stress was assessed by the levels of MDA and lipid ROS in TNBC cells treated with CM of control TAMs or IL-6 activated TAMs.

**h** EDU analysis of TNBC cells treated with CM of control TAMs, IL-6 activated TAMs alone or with LY2109761.

**i** Migration analysis of TNBC cells treated with CM of control TAMs, IL-6 activated TAMs alone or with LY2109761.

**j** Invasion analysis of TNBC cells treated with CM of control TAMs, IL-6 activated TAMs alone or with LY2109761.

**k** TNBC cells treated with CM of control TAMs, IL-6 activated TAMs alone or with LY2109761, and then treated with cisplatin with a dose escalation from 0 to 100 μg/ml.

**l** The indicated cells were treated with cisplatin (4 µg/ml) for 48 hours, and then subjected to flow cytometry analysis.

All results are presented as the mean ± SD, and statistical significance was assessed using a two-tailed Student t test. *p < 0.05.
