# Supplementary material for: HLF regulates ferroptosis, development and chemoresistance of triple-negative breast cancer by activating tumor cell-macrophage crosstalk
Source: J Hematol Oncol. 2022 Jan 6;15:2. doi: 10.1186/s13045-021-01223-x (PMC8740349; doi:10.1186/s13045-021-01223-x)
Supplement: Supplementary file 2 — Additional file 2: Supplementary Methods. [file 13045_2021_1223_MOESM2_ESM.doc]

**Additional file 2**

**Methods**

***Cell lines and viruses***

The TNBC cell lines MDA-MB-231 (MB-231) and HCC1937, the human monocyte cell line THP-1 were obtained from Cell Bank of Type Culture Collection of Chinese Academy of Sciences (Shanghai Institute of Cell Biology of the Chinese Academy of Sciences). HLF knockdown and control lentiviruses (designated as shHLF and GFP) were purchased from Cyagen (Guangzhou, China). The RNAi target sequence was as follows: GCTGGGCAAATGCAAGAACAT. GGT1 knockdown and control lentiviruses (designated as shGGT1 and GFP) were purchased from GenePharma (Shanghai, China). FLAG-HLF, FLAG-GGT1 or control lentiviruses (designated as HLF, GGT1 and Control) and Adeno-associated virus (AAV) targeting HLF or GFP (designated as AAV-shHLF and AAV-GFP) were obtained from Obio Technology Co. (Shanghai, China). The anti-Flag antibody was used to recognize exogenous Flag-tagged HLF or Flag-tagged GGT1 protein in Flag-tagged HLF or Flag-tagged GGT1 infected cells.

***Tissue microarrays (TMA)***

High-density tissue microarrays (TMA) of human TNBC clinical samples (Cat. no. BRC1601) were obtained from a cohort of 80 patients and constructed by Superbiotek Inc. (Shanghai, China). Detailed clinicopathological features of the patients are described in Additional file 3: Table S1. The use of the clinical samples in the project was approved by the Naval Military Medical University of Medicine Ethical Committee. The tissue samples were fixed with neutral buffered formalin and embedded in paraffin, followed by H&E staining or immunohistochemistry examination as described previously. The antibodies used are listed in Additional file 3: Table S2. The stained sections were further quantified and scored blindly by a pathologist. Additional details are provided in the Supplemental Data.

***Luciferase reporter assay***

The DNA sequences containing the promoter region of HLF (-292 - +109), GGT1 (˗2098 - ˗1655), IL-6 (˗350 - ˗50) and TGF-β1 (˗1050 - ˗750) were cloned into pGL6-luc plasmid (designated as HLF-WT and GGT1-WT). The potential SMAD3 binding sites within the HLF promoter, the potential HLF binding sites within the GGT1 promoter or IL-6 promoter, and the potential STAT3 binding sites within the TGF-β1 promoter were further deleted in pGL6-HLF-WT-luc, pGL6-GGT1-WT-luc, pGL6-IL-6-WT-luc and pGL6- TGF-β1-WT-luc plasmid, respectively (designated as HLF-Mut, GGT1-Mut, IL-6-Mut and TGF-β1-Mut). The cells were transfected with pGL6-HLF-WT-luc, pGL6-HLF-Mut-luc, pGL6-GGT1-WT-luc, pGL6-GGT1-Mut-luc, pGL6-IL-6-WT-luc, pGL6- TGF-β1-WT-luc, pGL6- TGF-β1-Mut-luc and pRL-TK-renilla-luc plasmids. Luciferase activity was measured using a Synergy 2 Multidetection Microplate Reader (BioTek Instruments, Inc.). Data were normalized for transfection efficiency by dividing firefly luciferase activity by *Renilla* luciferase activity.

***Chromatin immunoprecipitation (ChIP) assays***

The ChIP assay was performed utilizing an EpiTect ChIP qPCR Kit (QIAGEN) according to the manufacturer’s instructions. Briefly, the chromatin was immunoprecipitated with IgG, anti-SMAD3, anti-Flag, anti-STAT3(S727) or anti-STAT3(Y705) antibodies. Then, the DNA was purified, and qPCR was performed to examine the bound sequences. The primers used for qPCR analysis were listed in Additional file 3: Table S3.

***Flow cytometric analysis***

The apoptosis of TNBC cells was measured by flow cytometry using APC Annexin V Apoptosis Detection Kit (Biolegend B266205). Briefly, 1×10^6^ cells were harvested and washed twice with cold cell staining buffer, resuspended in 100 μL Annexin V binding buffer, then incubated with 5 μL of APC Annexin V and 5 μL of 7-AAD viability staining solution for 15 minutes at room temperature in the dark. The cell suspension was then incubated with 400 μL of Annexin V binding buffer followed by flow cytometry analysis.

***RNA interference***

Small interference RNAs (siRNAs) against SOX2, OCT4, STAT3, SMAD3, or GGT1 and NC (NC, negative control) siRNA were synthetized by Genepharma (Shanghai, China). siRNA target sequences are listed in Additional file 3: Table S4. The siRNAs were transfected into the TNBC cells at a final concentration of 200 nM using lip2000 according to the manufacturer’s instructions (Invitrogen, USA). The cells were harvested or subjected to further downstream experiments 24-72 hours after transfection.

***RNA-seq***

Total RNA (1 μg) was used for removing the rRNAs using Ribo-Zero rRNA Removal Kits (Illumina, San Diego, CA, USA) following the manufacturer's instructions. RNA libraries were constructed by using rRNA-depleted RNAs with TruSeq Stranded Total RNA Library Prep Kit (Illumina, San Diego, CA, USA) according to the manufacturer’s instructions. Libraries were controlled for quality and quantified using the BioAnalyzer 2100 system (Agilent Technologies, Inc., USA). 10 pM libraries were denatured as single-stranded DNA molecules, captured on Illumina flow cells, amplified in situ as clusters and finally sequenced for 150 cycles on Illumina HiSeq Sequencer according to the manufacturer’s instructions. High throughput sequencing service was provided by CloudSeq Biotech (Shanghai, China).

***ChIp-seq***

Chromatin Immunoprecipitation was performed according to Wamstad et al. (2012) (See Additional file 1: Method below for detail). The yield of ChIPed DNA was determined via Quant IT fluorescence assay (Life Technologies) and enrichment efficiencies of ChIP reactions were evaluated by qPCR. Illumina sequencing libraries were generated with NEBNext® Ultra™ DNA Library Prep Kit (New England Biolabs) by following the manufacturer’s manual. The library quality was determined by using Agilent 2100 Bioanalyzer (Agilent), and then, subjected to high-throughput 150 base paired-end sequencing on Illumina Hiseq sequencer according to the manufacturer’s recommended protocol. High throughput sequencing service was provided by CloudSeq Biotech (Shanghai, China).

***Western blot analysis***

Western blot analysis was performed as previously described [1]. The primary antibodies used are listed in Additional file 3: Table S2.

***Real-time PCR analysis***

Total RNA was isolated from cells or tissues using TRIZOL (Invitrogen) according to the manufacturer’s instructions. The purity of RNA was measured with a UV spectrophotometer (NanoDrop ND-1000) and RNA integrity was validated with agarose gel electrophoresis. The extracted RNA was then reverse-transcribed to cDNA with the M-MLV RTase cDNA Synthesis Kit (Promega). Real-time PCR analysis was performed using a SYBR Green PCR Kit (Roche) and LightCycler 480 System (Roche). PCR conditions included 1 cycle at 95 °C for 5 minutes, followed by up to 40 cycles of 95 °C for 15 seconds (denaturation), 60 °C for 30 seconds (annealing) and 72 °C for 30 seconds (extension). The sequences of primers used were listed in Additional file 3: Table S3. The specificity of primers was confirmed by melting curves following the reaction. Each sample was measured in triplicate biological replicates. Each experiment was repeated at least three times and the representative results were shown. The relative mRNA expression levels of certain genes were further presented as fold changes of gene expression in experiment group relative to control group.

***Cell proliferation and Colony growth assay***

For cell proliferation assay, the TNBC cells were seeded in 96-well plates (3×10^3^ cells per well). ATP activity was examined using a Cell Counting Kit-8 at the indicated time points. EdU staining was performed with the EdU Kit (RiboBio) following the manufacturer's instructions. The Edu^+^ cells are presented as the mean number of cells per field of view. For colony growth assay, the TNBC cells were seeded in 12-well plates (2×10^3^ cells per well) in triplicate and cultured for 7 days. The cells were then fixed with 10% formalin and stained with 0.5% crystal violet solution for 15 min. After staining, the wells were washed three times with PBS and pictured. The colonies formed were further examined and counted macroscopically. The colony counts were presented as the mean number of colonies per field of view. Each experiment was repeated at least three times. The representative results and images from a single experiment were shown.

***Cell migration and invasion assays***

For cell migration analysis, 2×10^5^ cells were seeded into the upper chamber of a polycarbonate transwell in serum-free DMEM. DMEM containing 20% fetal bovine serum as chemoattractant was added in the lower chamber. After 6 hours of incubation, the chamber was then fixed and stained. The cell counts were expressed as the mean number of cells per field of view. For the cell invasion assay, 2×10^5^ cells were seeded into the matrigel-coated chamber in serum-free DMEM. DMEM containing 20% fetal bovine serum as chemoattractant was added in the lower chamber. After 12 hours of incubation, the chamber was then fixed and stained. The cell counts were expressed as the mean number of cells per field of view.

***Primary Human TAMs Isolation from Breast Cancer***

TAMs were isolated from fresh breast cancer samples as previously described [2], with slight modifications. Briefly, the tissues were minced into small (1 to 2 mm) pieces and digested with 5% fetal bovine serum Dulbecco’s modified Eagle’s medium containing 2 mg/ml collagenase I and 2 mg/ml hyaluronidase (Sigma) at 37 ℃ for 2 hr. The cells were sequentially filtered through 500 mm mesh, 100 mm, and 70 mm cell strainer. The cells were then centrifuged in a Beckman Allegra X-15R centrifuge at 2,500 rpm for 20 min with 1 ml cell suspension above 5ml 45% Percoll (GE Healthcare) in the middle and 5ml 60% Percoll at the bottom in a 15 ml tube. Mononuclear cells were collected from the cell layer in the interphase between 45% and 60% Percoll. CD14^+^ monocytes and macrophages were isolated by a magnetic-activated cell sorting using direct CD14 Isolation Kit (Miltenyi Biotec) according to the manufacturer’s instructions.

***Iron assays***

Iron Assay Kit (Sigma-Aldrich, St. Louis, MO, USA) was executed to detect ferrous iron (Fe2+) or total iron in TNBC cells, in accordance with the operating procedure supplied by the producer and the previous description [3].

***Malondialdehyde (MDA) detection***

The relative MDA level was measured employing a Lipid Peroxidation Assay Kit (Beyotime). In concision, 0.2 mL MDA detection working solution was pipetted to 0.1 mL cell supernatant lysates of TNBC cells to mix thoroughly and heat for 15 min at 100℃, then the tube was centrifuged for 10 min at 1000 g after cooling down to room temperature. Then 200 μL supernatant was transferred into a 96-well plate and the absorbance of 532 nm was examined via a microplate reader.

***Lipid reactive oxygen species (ROS) detection***

Lipid ROS concentration was evaluated by staining of C11-BODIPY. 10 μM C11-BODIPY (Invitrogen) was added to the culture medium of TNBC cells in the six-well plates to incubate for 30 min away from light in the 37℃ incubator. The washed cells were resuspended in 500 μL phosphate buffer solution (PBS; Corning Inc.) and cell suspension was subjected to the detection of fluorescence intensity on a flow cytometer (BD Biosciences), followed by the calculation of relative ROS level.

***GSH/GSSG ratio measurement***

GSH/GSSG ratio were determined using the GSH and GSSG Assay Kit (Beyotime, S0053) following the manufacturer’s protocol.

***GPX4-Specific Activity***

TNBC cells were incubated for 5 min at room temperature in 1 mL of 0.1 M KH2PO4/K2HPO4, pH 7.8 containing 5 mM EDTA, 5 mM GSH, 0.1% (v/v) Triton X-100, 160 mMNADPH/H+ and 180 IU/ml glutathione reductase (GR). Enzymatic activity was triggered by adding 25 mM phosphatidylcholine hydroperoxide (PCOOH) and quantified as the decrease of absorbance at 340 nm due to NADPH/H+ oxidation by GR, as reported (Roveri et al., 1994). GPX4-specific activity was expressed as nmoles/min/mg.

***Xenografted tumor formation***

For in vivo tumor growth assay, TNBC cells were injected subcutaneously into six nude mice at 1×10^6^ cells per mouse. Xenografted tumor formation was monitored and the mice were sacrificed six weeks post-inoculation.

For pulmonary metastasis assay, TNBC cells (1×10^6^) were injected into the tail vein of nude mice (n=7). The mice were sacrificed 12 weeks post inoculation and consecutive sections of the whole lung were subjected to hematoxylin and eosin (H&E) staining. All metastatic foci in the lung were calculated microscopically to evaluate the development of pulmonary metastasis.

***Data analysis***

Statistical analysis was performed using SPSS 16.0 software (SPSS Inc., USA). The data are expressed as the mean ± SD. The significance of mean values between two groups was analyzed by Student’s t-test or Mann-Whitney U test. Pearson’s correlation analysis was performed to determine the correlati1on between two variables. A p-value less than 0.05 was considered statistically significant.

**Reference**

1. Zhou T, Li S, Xiang D, Liu J, Sun W, Cui X, Ning B, Li X, Cheng Z, Jiang W, et al. m6A RNA methylation-mediated HNF3γ reduction renders hepatocellular carcinoma dedifferentiation and sorafenib resistance. Signal Transduct Target Ther. 2020 12 26;5(1).

2. Kuang DM, Wu Y, Chen N, Cheng J, Zhuang SM, Zheng L. Tumor-derived hyaluronan induces formation of immunosuppressive macrophages through transient early activation of monocytes. Blood. 2007 Jul 15;110(2)

3. Kim DH, Kim WD, Kim SK, Moon DH, Lee SJ. TGF-beta1-mediated repression of SLC7A11 drives vulnerability to GPX4 inhibition in hepatocellular carcinoma cells. Cell Death Dis. 2020;11:406.
