# Supplementary material for: HLF regulates ferroptosis, development and chemoresistance of triple-negative breast cancer by activating tumor cell-macrophage crosstalk
Source: J Hematol Oncol. 2022 Jan 6;15:2. doi: 10.1186/s13045-021-01223-x (PMC8740349; doi:10.1186/s13045-021-01223-x)
Supplement: Supplementary file 3 — Additional file 3: Supplementary Tables. [file 13045_2021_1223_MOESM3_ESM.doc]

**Additional file 3: Tables**

**Table S1. Clinicopathological features of 80 NTBC patients**

| Characteristics |  |  | HLF low  (n=40) | HLF high  (n=40) | p value |
| --- | --- | --- | --- | --- | --- |
| Age(year) | ≤50 |  | 20 | 21 | >0.05 |
|  | >50 | | 20 | 19 |  |
| Tumor size(cm) | ≤2 | | 27 | 6 | <0.05 |
|  | >2 | | 13 | 34 |  |
| Lymph node metastasis | No | | 29 | 13 | <0.05 |
|  | Yes | | 11 | 27 |  |
| TNM | I-II |  | 35 | 19 | <0.05 |
|  | III-IV |  | 5 | 21 |  |

TNM, Tumor-Nodes-Metastasis.

**Table S2.** Antibody List.

| **Antigens** | **Manufacturer** | **Application** |
| --- | --- | --- |
| HLF | Abcam ab91630, USA | 1:1000 for WB |
| HLF | The International Cooperation Laboratory on Signal Transduction, EHBH, SMMU, China | 1:100 for IHC or immunofluorescence staining (IF) |
| GGT1 | Signalway Antibody, USA | 1:1000 for WB or 1:100 for IHC |
| GGT1 | Abcam, USA | 1:50 for IF |
| SMAD3 | Abcam, Cambridge, MA | 1:1000 for WB,  1:100 for Chip or 1:50 for IF |
| Flag | Abcam, Cambridge, MA | 1:1000 for WB or  1:100 for Chip |
| Ki67 | Proteintech Group, China | 1:100 for IHC |
| PARP | Proteintech Group, China | 1:1000 for WB |
| GAPDH | Santa Cruz Biotechnology, CA | 1:5000 for WB |
| CD4 | Abcam, Cambridge, MA | 1:50 for FACS |
| CD8 | Proteintech Group, China | 1:50 for FACS |
| F4/80 | Abcam, Cambridge, MA | 1:50 for FACS |
| CD9 | Proteintech Group, China | 1:500 for WB |
| CD14 | Abcam, Cambridge, MA | 1:50 for FACS |

**Table S3. P**rimer List.

| **Gene** | **Forward primer (5’-3’)** | **Reverse primer (5’-3’)** | |
| --- | --- | --- | --- |
| HLF(Human) | Forward (5*′*- 3*′*) | ACCAAGTCCCATTGATCCTG | |
|  | Reverse (5*′*- 3*′*) | GCCCAGTACTTGTCATCCTTC | |
| GGT1(Human) | Forward (5*′*- 3*′*) | ATCTTAGCTTGGTTTTGGT | |
|  | Reverse (5*′*- 3*′*) | ACCTATGATGTCCACCAG | |
| β-actin(Human) | Forward (5*′*- 3*′*) | GGCCCAGAATGCAGTTCGCCTT | |
|  | Reverse (5*′*- 3*′*) | AATGGCACCCTGCTCACGCA | |
| CHIC2(Human) | Forward (5*′*- 3*′*) | CACATTAGGTTGCAGTATGT | |
|  | Reverse (5*′*- 3*′*) | TATTCCATCATGTTATTCGT | |
| CYS1(Human) | Forward (5*′*- 3*′*) | AGAAGTCACAGGAATTGTTC | |
|  | Reverse (5*′*- 3*′*) | TTATTTGGCTAGCTGCTATT | |
| FAM177B(Human) | Forward (5*′*- 3*′*) | ATCTATTGAACAATTTCTAACATC | |
|  | Reverse (5*′*- 3*′*) | AAACAAAACAAATAATTCCTCT | |
| PER3(Human) | Forward (5*′*- 3*′*) | | GTGGTGAATGTAAGACCTTT |
|  | Reverse (5*′*- 3*′*) | | AATGTTGTAGCTCTTCAGGT |
| PLA2G4C(Human) | Forward (5*′*- 3*′*) | | ATGGGAAGAAAGAAGAAAA |
|  | Reverse (5*′*- 3*′*) | | TTAGCTTCTTCAGAGCTTTC |
| PTHLH(Human) | Forward (5*′*- 3*′*) | | AGCGTGTGAACATTCCT |
|  | Reverse (5*′*- 3*′*) | | CTGAGAACAAGTTTCAAGTG |
| PTPN22(Human) | Forward (5*′*- 3*′*) | | ATGAAGTCAGTAAACCACAG |
|  | Reverse (5*′*- 3*′*) | | GCAGAGCAAGAAAAATAGTC |
| SLC22A15(Human) | Forward (5*′*- 3*′*) | | GAGCTTTACCCTACAGTCAT |
|  | Reverse (5*′*- 3*′*) | | GATGAAGGGAGCAATAATC |
| HIST1H4B(Human) | Forward (5*′*- 3*′*) | | GGTTTGATTTATGAGGAGAC |
|  | Reverse (5*′*- 3*′*) | | AGAAAACTGACGAAAAGATT |
| KCNJ1(Human) | Forward (5*′*- 3*′*) | | ACACAAATTAGCGTCAGTC |
|  | Reverse (5*′*- 3*′*) | | CTGGTTGTTGGTCTTTCTAT |
| MCTP1(Human) | Forward (5*′*- 3*′*) | | GATTTAGCAGGTGAAGTTTT |
|  | Reverse (5*′*- 3*′*) | | CCATTGCAAAACTGTTAGTA |
| NFIL3(Human) | Forward (5*′*- 3*′*) | | TGCTCAGTCGGCAAC |
|  | Reverse (5*′*- 3*′*) | | AGCTCTTTAAAAACTCTGGTT |
| USP29(Human) | Forward (5*′*- 3*′*) | | GAAGTGTGGTCCTTAGACAT |
|  | Reverse (5*′*- 3*′*) | | GTCCAGGAACATATTCAACT |
| ZSCAN31(Human) | Forward (5*′*- 3*′*) | | AGCTCTCCAAATGAGACTT |
|  | Reverse (5*′*- 3*′*) | | TTAATTTGGAAGGCTTACTC |
| PAK6(Human) | Forward (5*′*- 3*′*) | | ACCCCAAAACCCTATCT |
|  | Reverse (5*′*- 3*′*) | | CAGACACCTGAAGTTCTTTC |
| Mrc1(Human) | Forward (5*′*- 3*′*) | | ACCTGCGACAGTAAACGAGG |
|  | Reverse (5*′*- 3*′*) | | TGTCTCCGCTTCATGCCATT |
| Cd163(Human) | Forward (5*′*- 3*′*) | | GAAGACAGAGACAGCGGCTT |
|  | Reverse (5*′*- 3*′*) | | GGTATCTTAAAGGCTCACTGGGT |
| Stab1(Human) | Forward (5*′*- 3*′*) | | TGGCCCCAGGGACAGTT |
|  | Reverse (5*′*- 3*′*) | | TGGCCAGAGCATGGATGATG |
| Il10 (Human) | Forward (5*′*- 3*′*) | | TTGCCTGGTCCTCCTGACTG |
|  | Reverse (5*′*- 3*′*) | | TCGGAGATCTCGAAGCATGT |
| Nos2(Human) | Forward (5*′*- 3*′*) | | CGCATGACCTTGGTGTTTGG |
|  | Reverse (5*′*- 3*′*) | | CATAGACCTTGGGCTTGCCA |
| Arg1(Human) | Forward (5*′*- 3*′*) | | ACTTAAAGAACAAGAGTGTGATGTG |
|  | Reverse (5*′*- 3*′*) | | ATTGCCAAACTGTGGTCTCC |
| TGF-β1(Human) | Forward (5*′*- 3*′*) | | CTGTCCAACATGATCGTGCG |
|  | Reverse (5*′*- 3*′*) | | GACACAGAGATCCGCAGTCC |
| IL-6(Human) | Forward (5*′*- 3*′*) | | TGATGCACTTGCAGAAAACA |
|  | Reverse (5*′*- 3*′*) | | TCCTCGTTGTGGGAGGTGAT |
| IL-13(Human) | Forward (5*′*- 3*′*) | | GGGATAAGGGGCGTTGACTC |
|  | Reverse (5*′*- 3*′*) | | AGCTGTCAGGTTGATGCTCC |
| PDGF-BB(Human) | Forward (5*′*- 3*′*) | | GCGCCCATTTTTCATTCCCT |
|  | Reverse (5*′*- 3*′*) | | CCGGTTTTCTCTTTGCAGCG |
| FGF-6(Human) | Forward (5*′*- 3*′*) | | GCGTGGTGAGTCTCTTTGGA |
|  | Reverse (5*′*- 3*′*) | | ACCCGTCCGTATTTGCTCAG |
| IGFBP-1(Human) | Forward (5*′*- 3*′*) | | TGGGACGCCATCAGTACCTA |
|  | Reverse (5*′*- 3*′*) | | CTCCTGATGTCTCCTGTGCC |
| PARC(Human) | Forward (5*′*- 3*′*) | | CTTGTCCTCGTCTGCACCAT |
|  | Reverse (5*′*- 3*′*) | | CTGGGGGCTGGTTTCAGAAT |
| GGT1 ChIP | Forward (5*′*- 3*′*) | | AAGGGTGTGAGCTGTCCAACG |
|  | Reverse (5*′*- 3*′*) | | TCCAACATCACCTGCCCTTCA |
| HLF ChIP | Forward (5*′*- 3*′*) | | TTTCTCATTCCTCTTCCCACCCA |
|  | Reverse (5*′*- 3*′*) | | TTCGGGGACCTTGCAGCAC |
| IL-6 ChIP | Forward (5*′*- 3*′*) | | GCCTCAATGACGACCTAAGC |
|  | Reverse (5*′*- 3*′*) | | TCATGGGAAAATCCCACATT |
| TGF-β1 ChIP | Forward (5*′*- 3*′*) | | AGTATCAGGGAGTGGGGAATCAG |
|  | Reverse (5*′*- 3*′*) | | TACTGGGCACATGGCAAAATCC |
| siHLF | 5’-UGGGCAAAUGCAAGAACAUTT-3’ | | |
| siSTAT3 | 5’-CCACUUUGGUGUUUCAUAATT-3’ | | |
| siSMAD3 | 5’-GGCCAACAUAGGCAAAUGATT-3’ | | |
| siOCT4 | 5’-AACAUGUGUAAGCUGCGGCCCdTdT-3’ | | |
| siSOX2 | 5’-CCAUGGAUUUAUUCCUAAATT-3’ | | |
| siTGF-β1 | 5’-GCAACAACGCCAUCUAUGA-3’ | | |
